# Pharmaceutical Cocrystals: New Solid Phase Modification Approaches for the Formulation of APIs

**DOI:** 10.3390/pharmaceutics10010018

**Published:** 2018-01-25

**Authors:** Anna Karagianni, Maria Malamatari, Kyriakos Kachrimanis

**Affiliations:** 1 Department of Pharmaceutical Technology, Aristotle University of Thessaloniki, 54351 Thessaloniki, Greece; karagiak@pharm.auth.gr; 2 Faculty of Engineering and Science, University of Greenwich at Medway, Kent ME4 4TB, UK; M.Malamatari@greenwich.ac.uk

**Keywords:** cocrystals, coformers, solid state properties, poorly soluble APIs

## Abstract

Cocrystals can be used as an alternative approach based on crystal engineering to enhance specific physicochemical and biopharmaceutical properties of active pharmaceutical ingredients (APIs) when the approaches to salt or polymorph formation do not meet the expected targets. In this article, an overview of pharmaceutical cocrystals will be presented, with an emphasis on the intermolecular interactions in cocrystals and the methods for their preparation. Furthermore, cocrystals of direct pharmaceutical interest, along with their in vitro properties and available in vivo data and characterization techniques are discussed, highlighting the potential of cocrystals as an attractive route for drug development.

## 1. Introduction

When determining the physical form in which active pharmaceutical ingredients (APIs) will be administered, the optimization of properties including solubility, dissolution rate, mechanical properties, hygroscopicity, physical stability and chemical stability is of strategic importance. Most APIs are solid, and exist in crystalline form, since these tend to be more stable, reproducible in their properties, and more easily isolated in high purity than the amorphous drugs and formulations. Nevertheless, a great number—40%—of commercial compounds and drugs under development, and 80% of the drug substances that are in the production line, appear to have solubility problems [1]. The oral absorption of drugs with high permeability but low solubility (class II of the biopharmaceutics classification system, BCS) is limited due to their poor solubility. Therefore, the increasing number of drug substances suffering from poor solubility is one of the most common issues hindering drug development [2].

**Figure 1:**
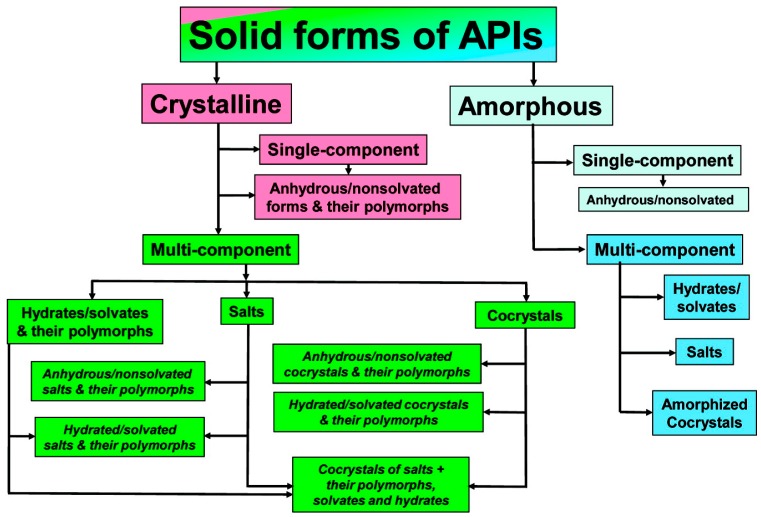
API solid form classification based on structure and composition. (Reprinted from [4] with permission. Copyright 2012 American Chemical Society).

**Figure 2:**
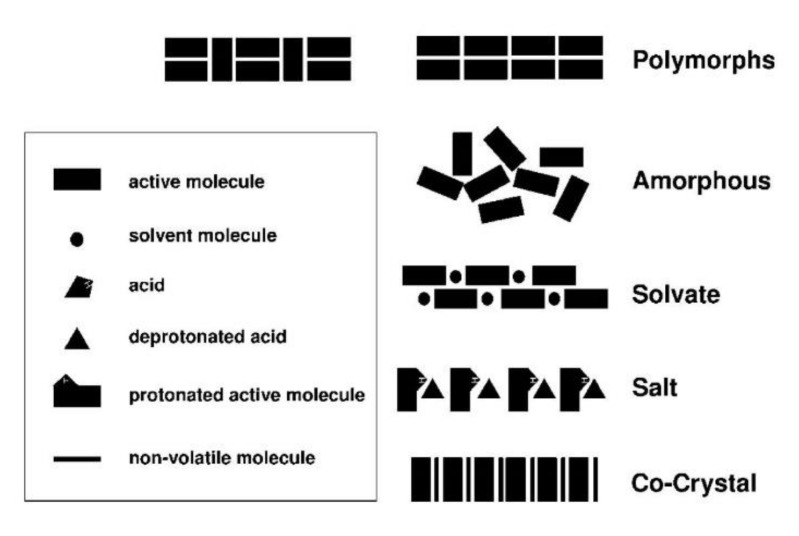
Schematic representation of the structural relationship between the amorphous state, polymorphs, solvates-hydrates, salts and cocrystals (Reprinted from [9] with permission. Copyright 2006 WILEY-VCH Verlag).

In order to improve the physicochemical properties of APIs, various solid-phase modifications have been reported (Figure 1 and Figure 2). In addition to the amorphous state, these include alternate crystalline structures such as polymorphs, salts, solvates, hydrates, and recently, pharmaceutical cocrystals, which can be used as an alternative approach to enhancing specific physical properties of pharmaceutical ingredients [3].

Amorphicity is defined with reference to crystallinity. Similar to crystallinity, short-range molecular order (i.e., in terms of the relationships between neighboring molecules) may exist in amorphous solids, but there is no long-range order of molecular packing or well-defined molecular conformation. For pharmaceutical materials, amorphicity offers advantages, since amorphous solids have higher solubility, higher dissolution rate, and sometimes better compression characteristics than the corresponding crystals. However, the amorphous state is not thermodynamically stable, and this leads to higher physical and chemical instability compared to the crystalline state [5]. According to the European Pharmacopoeia, polymorphism is the property whereby a substance is able to form different crystalline forms that are phases with different crystalline structures of a single component. Polymorphs have the same chemical composition, but different physicochemical properties, due to the ability of atoms or molecules to be joined together in different ways, or to take different conformations in space [6]. These different polymorphs vary in terms of properties such as chemical stability and mechanical properties, which determine, for instance, ease of tableting, behavior during formulation, resistance to thermal and mechanical stress, and solubility and dissolution rate, which affects absorption and bioavailability. Solvates are formed by the incorporation of solvent molecules into the crystalline lattice of a compound and are considered as molecular complexes between host and solvent molecules. When the solvent is water, they are called hydrates, and are viable forms for drug products, as there are no safety concerns surrounding water as a crystal adduct. Depending on how the water molecules are incorporated into the crystal lattice, hydrates can be further divided into hydrates where water molecules exist at isolated sites, channel hydrates, and ion-coordinated site hydrates [7]. Approximately 1/3 of drug molecules can form hydrates from anhydrous crystalline forms through changes in temperature, pressure or relative humidity, which can result in significant changes in physical properties, and can create severe problems during storage, wherein the appearance and the integrity of the dosage form may be altered. Salt formation is common between acidic and basic or zwitter-ionic substances, and is a simple, cost-effective method for improving low water solubility and enhancing an API’s bioavailability. Salts can also increase an API’s purity, crystallinity, possibility of isolation, and stability, as well as various technological characteristics, such as flowability. It is believed that more than half of marketed drugs are administered as salts. As a rule of thumb, if the ΔpKa shift is greater than or equal to 3 between the components, then the substance is referred to as salt. Cocrystals are viable alternative solid forms when approaches based on salt or polymorph formations do not meet the required targets. A cocrystal is a crystalline entity-complex formed by two (or more) different, distinct molecular entities/ingredients, which are solid at ambient temperatures, and within which the intermolecular interactions in the unique crystal lattice generated are weak forces (non-covalent and non-ionic), such as hydrogen bonds, π bonds and van der Waals bonds. At this point, it should be mentioned that there is considerable debate surrounding the definition of cocrystal. According to the US Food and Drug Administration (FDA) Directive (2013), cocrystals are defined as “solids which are crystalline materials composed of two or more molecules in the same crystal lattice” [8]. Another generally accepted definition of pharmaceutical cocrystals, which was coined in the context of the Indo-US Bilateral Meeting on the Evolving Role of Solid State Chemistry in Pharmaceutical Science (India, February 2012), is the following: “Cocrystals are solids that are crystalline single phase materials composed of two or more different molecular and/or ionic compounds generally in a stoichiometric ratio which are neither solvates nor simple salts” [4].

In this article, an overview of pharmaceutical cocrystals will be presented, with an emphasis on the intermolecular interactions in cocrystals, and the methods that have been used for their production. Moreover, cocrystals of pharmaceutical interest, their in vitro properties, and any available data on their in vivo performance will be discussed. The final section of this review covers, in brief, techniques for the characterization of cocrystals, especially those that are routinely used in drug delivery and development laboratories.

## 2. History and Definition of Cocrystals

**Figure 3:**
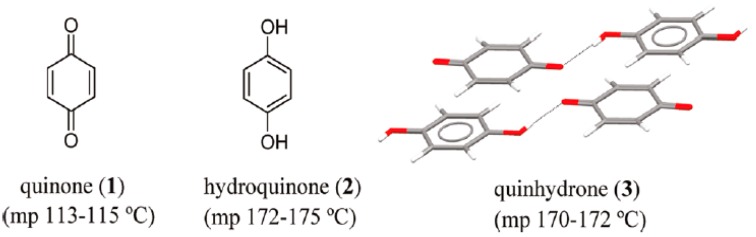
Structures of quinine, hydroquinone, and quinhydrone. (Reprinted from [11] with permission. Copyright 2009 American Chemical Society).

Cocrystals’ history begins in 1844 with Friedrich Wohler and the discovery of the first cocrystal, quinhydrone, during the study of quinones [10]. This cocrystal (back then it was not identified as such, since X-ray analysis was not available, and it was only in 1958 that its complete structure and intermolecular interactions were published) consists of quinone and hydroquinone in a ratio of 1:1, see Figure 3 [11].

In fact, many of the first cocrystals were hidden under different names, such as adducts, molecular complexes, organic molecular compounds and solid state complexes. Many were discovered in the early 1900s. According to Paul Pfeiffer (1922) in his book “Organische Molekulverbindungen”, cocrystals are divided to those consisting of both inorganic and organic components, and to those composed of organic components only [12].

Although the first cocrystal patent dates back to 1937 [13], the term “cocrystal” was not used until 1967, when it was suggested to describe a complex of hydrogen bonds that is formed between 9-methyl adenine and 1-methyl thymine. The term was subsequently spread in the 1990s by Margret Etter [14]. The debate on cocrystals began in 2003 with a controversial letter by Desiraju explaining his preference for it to be known as “a multi-component system held together by non-covalent interactions” [15]. A reply came from Dunitz, who pointed out that the term included solid solutions, encapsulated compounds or amorphous solids [16]. Aakeroy, for his part, poposed strict compliance with three criteria for the definition of a cocrystal [17]:

(1) The neutrality of the ingredients,
(2) The solid state of the components in ambient conditions, and
(3) The homogeneity of the crystalline material and the stoichiometry of the components.

Disagreement comes from Andrew Bond with regard to criterion 2, and he suggests the term “multi-component molecular crystals” to describe a crystalline material whose components are either solid or liquid in ambient conditions [18]. The FDA Directive proposed an adequate definition of the cocrystal, but a new field of debate has arisen as to whether cocrystals should be considered therapeutically equivalent, as well as with regard to the necessary toxicity and efficacy studies.

Compared with pharmaceutical salts, cocrystals have the following advantages: In theory all types of molecules can form cocrystals (weakly-ionizable and non-ionizable APIs), even though they may have limited or no ability to form salts. Furthermore, there are more possible options when choosing molecules to be used in the composition of cocrystals (coformers). These are substances that are on the FDA’s list of Generally Recognized As Safe (GRAS) substances, while for toxicological reasons less acidic or basic counter ions are typically used in a salt API (there are about 12 that are frequently used in the market and which are regarded as pharmaceutically acceptable) [19,20].

A cocrystal is a homogeneous crystalline phase with a well-defined stoichiometric ratio, e.g., 1:1, 1:2, etc. The existence of typical donors of hydrogen bonds, e.g., carboxylic acids, and receptors, e.g., amine or amide, are important factors for cocrystal formation. The best donor (d. Η) and the best receiver (r. H) will preferably form hydrogen bonds with each other. In terms of their differentiation from salts, the FDA Directive suggests that “traditionally, solid state pharmaceutical forms of APIs are grouped either as polymorphs or as salts”. Cocrystals, however, are distinct forms of these conventional pharmaceutical solid forms. Unlike polymorphs, which contain only one API in the crystal lattice, cocrystals are composed of an API with a neutral molecule (coformer compound) in the crystal lattice. Unlike salts, where the components of the crystal lattice are in an ionized state, the cocrystals’ components are in a neutral state and interact through non-ionic interactions. Hence, one difference between salts and cocrystals is that in salt formations there is a proton transfer and ionization, while these do not appear in the cocrystals. Cocrystals are considered to be formed when Δp*K*a < 2, and are ultimately regarded as molecular “API-excipient” complexes able to stand the molecular bond taking place within the crystal lattice, thus constituting an “intermediate pharmaceutical product” [8]. However, the extent of proton transfer is known to be affected by both the crystal environment [21] and temperature [22], indicating that salts and cocrystals form a continuum, rather than having a clear borderline between them, which complicates their identification for regulatory purposes.

**Figure 4:**
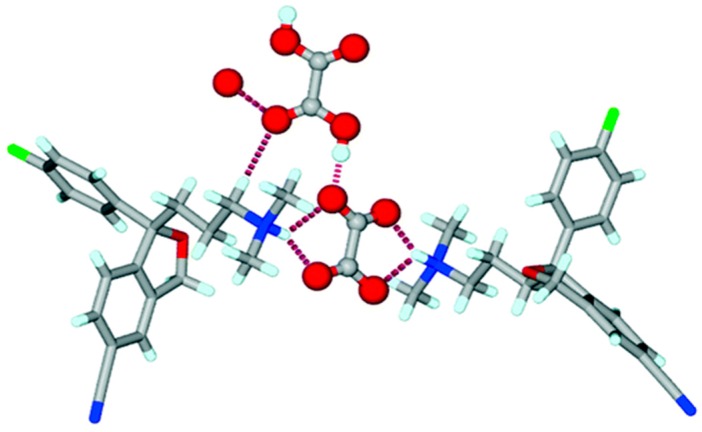
The crystal structure of escitalopram oxalate, a market drug (Depakote^®^, Depakine^®^), reveals the presence of protonated escitalopram cations that hydrogen bond to oxalate dianions, water molecules, and oxalic acid molecules in the same crystal. Grey: carbon; Green: fluorine; Blue: nitrogen; Red: oxygen. (Reprinted from [4] with permission. Copyright 2012 American Chemical Society).

The exact definitions of cocrystals and salts in the scientific community are sometimes ambiguous, e.g., escitalopram oxalate, which is considered a 1:1 salt between the base of escitalopram and oxalic acid, contains one oxalate dianion and a neutral oxalic acid molecule per two escitalopram cations in the crystal lattice. Thus, the oxalate di-escitalopram is cocrystalized with oxalic acid, and can behave either as a cocrystal or as a salt, see Figure 4 [23].

The European Medicines Agency (EMA) defines cocrystals as a variant of solid forms of APIs, associating them with salts, polymorphs, hydrates and solvates. In 2015, the EMA released a document specifically related to the use of cocrystals in pharmaceutical research, in addition to the one released in 2014 [24,25]. Nevertheless, cocrystals are expected to consist of two neutral components held together by non-covalent bonds, pointing out that there may be intermediate states between salts and cocrystals (salt cocrystals).

Polymorphism suggests that a compound, which may exist in other crystalline forms, will have a degree of conformation flexibility. Thus, the surface energy describing its thermodynamics and allowing it to grow into crystals offers a higher probability of forming cocrystals. Polymorphic compounds, as a result of their structural flexibility, are not energy-‘locked’ into a single type of crystal lattice or into a sealed structure. However, structural flexibility is not the only requirement, as the selection of molecules with alternative packing standards and synthon formation flexibility—that is, the ability to participate in different strong, well-defined intermolecular interactions—is equally important. At the same time, π bonds and van der Waals interactions are important, as well [26]. Therefore, in the beginning, there should be a study of the API on the number and the arrangement of the hydrogen bond donors and acceptors, the salt forming ability (p*K*a’s), the lattice energy, the conformation flexibility and solubility requirements, as well as the molecular weight (usually small). The appropriate coformer is often selected on the basis of the hydrogen bonding rules, the molecular recognition probability and the toxicological profile. To date, it has not been possible to fully predict whether a cocrystallizing reaction will be successful or not, and thus the reactions are carried out experimentally under different conditions, with different techniques to find cocrystals. Nevertheless, the possible intermolecular interactions in cocrystals and the computational predictive approaches and experimental screening techniques will be reviewed.

## 3. Intermolecular Interactions in Cocrystals

The high degree of interest in cocrystals is obvious from the increasing numbers of publications, applications for patents, and their recent introduction in the Cambridge Structural Database (CSD), which is an essential tool in the field of crystal engineering. The data collected in the database contribute to our understanding of the supramolecular interactions between functional groups within crystal structures. The analysis of cocrystals’ structures in the CSD shows that hydrogen bonds are the predominant form of interaction between cocrystal components; it also shows that patterns of hydrogen bonds called supramolecular synthons occur very frequently in the structures of cocrystals referred to as heterosynthons. The evaluation of synthons’ stability is based on the frequency of their formation, which is obtained from the possibility of their formation between all structures containing the necessary functional groups.

**Figure 5:**
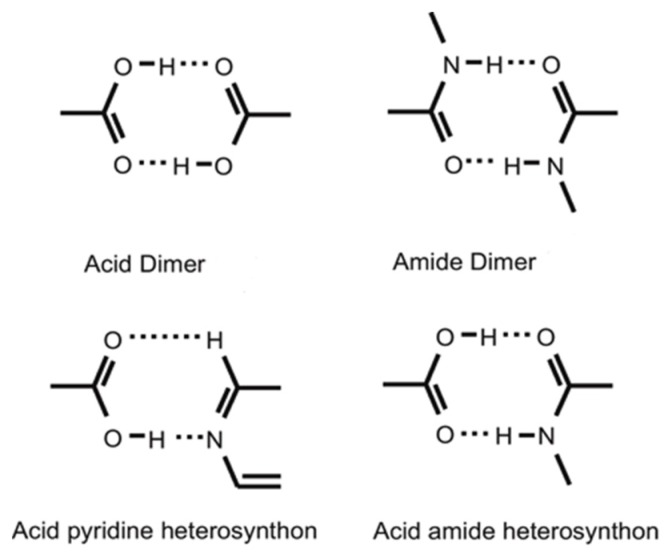
Common synthons found between carboxylic acid and amide functional groups (adapted from [30] with permission. Copyright 2013 Elsevier).

The term “synthon” was coined in 1967 by E. J. Corey [27], while Desiraju mainly used the term “supramolecular synthons” to mean “structural units in the supramolecule which can be formed or assembled by known or possible intra-/intermolecular interactions” when describing a series of cocrystals [28]. Supramolecular synthons, when added to a specific group of crystal structures, regulate their architecture (Figure 5). Bis et al. (2007) have conducted various studies aiming to discover homo- and heterosynthons, and have found that some supramolecular heterosynthons are strongly preferred to supramolecular homosynthons [29].

Based on a CSD statistical analysis [31], a series of instructions has been developed for selecting a favorable formation of hydrogen bonds in the crystal structure (hydrogen bonding rules):

(1) All good proton donors that are available in the molecule will be used for hydrogen bond formation in the crystalline structure of the compound.
(2) All good acceptors will be used for hydrogen bond formation, when there are available hydrogen bond donors.
(3) Intramolecular hydrogen bonds of six-member rings preferably form bimolecular hydrogen bonds.
(4) The best donor and the best acceptor of hydrogen bond will preferably form hydrogen bonds with one another [14]. Strong hydrogen bonds are N–H…O, O–H…O, N–H…N, and O–H…N, while weak hydrogen bonds include C–H…O–N and C–H…O=C, where a dash shows covalent and dots non-covalent bonds [32].

As cocrystals are generally viewed as one extreme on the salt-cocrystal “continuum”, which differ from salts only on the basis of the extent of proton transfer between an acid and a base [21], much focus has been placed on characterizing and possibly quantifying the relative strength of various hydrogen bond donor–acceptor supramolecular synthon interactions using charge density analysis [33]. More specifically, Hathwar et al. [34] compiled a library of multipolar parameters for supramolecular synthons that later proved to be transferable to a series of hydrogen-bonded cocrystal systems.

Dunitz and Gavezzotti [35] compared the absolute and relative stability of several supramolecular synthons with an emphasis on hydrogen bonds, but also including aromatic stacking and other types of interactions, such as C–H…O, Cl…Cl and C–H…Cl.

In a more recent study, Gavezzotti et al. [36] studied many cocrystals in the CSD, and concluded that 86% of known cocrystals are hydrogen-bonded. Among the hydrogen-bonded cocrystals, all known structures include a hydrogen bond between the coformers, while some also include hydrogen bonds between molecules of one coformer, and to a lesser extent all three possible combinations. The most commonly found hydrogen bond acceptors are carbonyl oxygens and aromatic nitrogens, while the hydrogen bond donors can be ranked in order of decreasing activity in the following order: COOH > NH >> R–OH. This makes the COOH···N (aromatic) the favorite hydrogen bond, most commonly found in cocrystals. The crystal packing is generally tight, with a packing coefficient of 0.75 and the lattice energy of the cocrystals is generally lower than the sum of the energies of the coformers. 

However, as bonding in crystals is nowadays viewed as non-localized, rather than merely a matter of short contacts between atoms in molecules, several other forms of interactions are considered significant, and many cases of cocrystals lacking hydrogen bonds in their structures have been identified. Non hydrogen-bonded cocrystals are based almost exclusively on π···π stacking between the two coformers, which are most commonly flat aromatic hydrocarbons. The main structural motifs in these cocrystals have been identified as the “alternate-ladder” and “slanted column” [37].

## 4. Prediction and Screening of Cocrystal Formation

**Figure 6:**
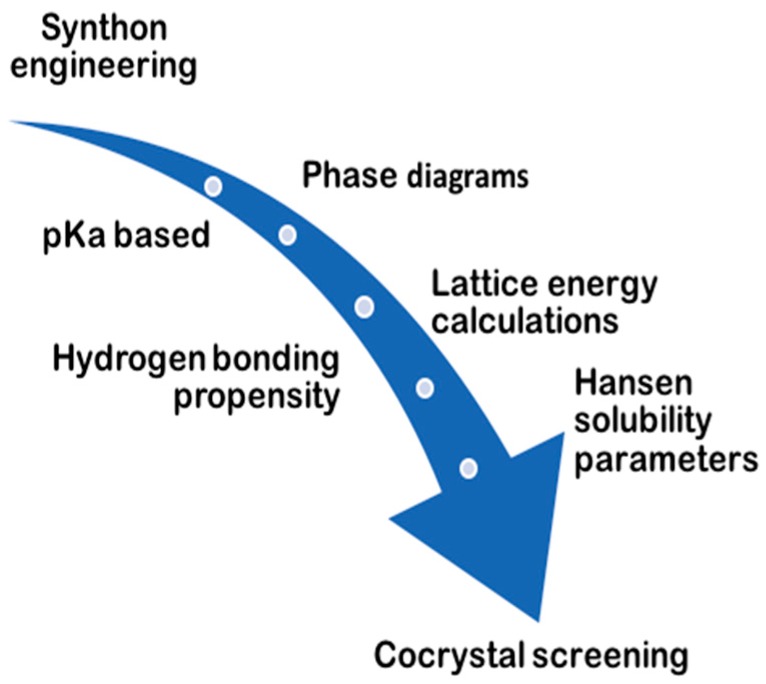
Approaches for cocrystal prediction and screening [44].

Various approaches have been applied in the prediction of cocrystal formation (Figure 6). A detailed review on experimental cocrystal screening and the role of phase diagrams has been provided by Malamatari et al. [38].

Computational predictions of cocrystal formation have been carried out by measuring the lattice energy under the crystal structure prediction (CSP), to confirm whether cocrystals are thermodynamically more stable than the pure crystals of the substances [39]. Another approach is based on the measurement of molecular electrostatic potential surfaces (MEPS) to assess molecular complementarity, as well as the possibility of forming cocrystals based on an electrostatic model that recognizes intramolecular interactions as a contact point between specific interaction points on the molecular surfaces [40].

Furthermore, with the systematic screening method, potential coformers are classified through information systems such as CSD; by targeting those that are able to form several patterns of hydrogen bonds with the API, the possibility of finding compounds suitable for cocrystal formation is increased. For instance, studies of CSD statistic have suggested that carboxylate and pyridine supramolecular heterosynthons are statistically more likely to occur than acid-acid supramolecular homosynthons [41]. The supramolecular heterosynthons that preferably appear in the formation of cocrystals have now been characterized, and include carboxylic acid-aromatic nitrogen, carboxylic acid-amide and alcohol-pyridine.

For the experimental screening of cocrystal formation, binary phase diagrams with the aid of thermal analysis and solubility phase diagrams (binary and ternary) have been used; while recently, several high-throughput methods have been reported [42,43].

## 5. Cocrystals of Direct Pharmaceutical Interest

The initial goal of pharmaceutical cocrystallization involved the modification of the properties of a drug molecule for the formation of pharmaceutical API cocrystals with an associated conformer, which would ultimately improve the properties compared to the crystalline structure of the original drug. This is due to the fact that physical properties depend on the molecular order in the solid form, and changes to and/or interactions between molecules usually have a direct effect on these properties. For this reason, publications viewing the field of cocrystals as a strategy for optimizing the physicochemical properties of the APIs have grown significantly over the past 25 years [45]. Over the last decade, pharmaceutical cocrystals have attracted a great deal of interest, following the successful phase II clinical study of a fixed-dose combination of celecoxib and tramadol by ESTEVE and Muldipharma Laboratories GmbH for severe pain relief. Hence, even the idea of developing multi-drug cocrystals holds great potential. Certainly, there is huge potential in researching the formation of cocrystals of existing APIs with one another in order to enhance the solubility and bioavailability of the product; but at the same time, ways must be found to increase the probability of success in creating cocrystals with two drugs. The studies on the theophylline-phenobarbital cocrystals in stoichiometry 2:1 or the meloxicam-aspirin cocrystals, which have been found to reduce the time needed to reach therapeutic concentration and accelerate the effect onset, are good examples [46,47]. Acetaminophen-theophylline cocrystals have a faster dissolution rate than physical mixtures [48]. The lamivudine-zidovudine cocrystals for the treatment of HIV infection offer higher therapeutic efficacy [49]. Drugs with similar structures and 3-D arrangements can be used for drug and drug synergy to obtain multi-drug cocrystal systems. Moreover, the development period for medications is reduced (including clinical tests), since cocrystals are not new chemical entities, and there is no structural modification of the APIs. Thus, they provide a unique opportunity to strengthen commercial formulations, but also to advance the development processes of substances that have not been developed due to low solubility or reduced stability.

The great interest in developing techniques for creating complex and resistant cocrystals (combinations of APIs or multi-drug cocrystal systems) is evidenced by the continuing increase in patent applications to the European Patent Office and the Patent Office of the United States to safeguard copyrights. At this point, there are formulations that, according to the FDA definition, can be categorized as cocrystals, including:

- Entresto^®^ (sacubitril-valsartan), which was approved by the FDA in 2015 for the treatment of heart failure. It is the first drug in a class combining valsartan (angiotensin receptor) and sacubitril (inhibitor neprilysinis) to reduce cardiovascular mortality [50].
- Lexapro^®^ (escitalopram oxalate), which was approved in 2009 for the treatment of depression and anxiety. It belongs to the selective serotonin reuptake inhibitors [23].
- Depakote^®^ (valproate sodium cocrystal with valproic acid). It is used to treat seizure disorders, manic depression, and to prevent disorders [51].

Despite their promising development, the application of cocrystals in the pharmaceutical industry is still limited due to the lack of a suitable production method on a large scale, as well as the uncertainty created by their classification by FDA as “intermediate medicinal products”, regarding the coformer as an excipient.

## 6. Drug Properties That Can Be Altered by Cocrystallization

Typical examples of in vitro (physicochemical) and in vivo (biopharmaceutical) properties of an API that can be set through cocrystallization include the following:

(1) **Melting point (m.p.)**: Melting point is a fundamental physical property, and is defined as the temperature at which the solid phase is in equilibrium with the liquid phase, and it is a thermodynamic process in which the free transition energy is zero. High m.p. is usually desirable, but may contribute to low solubility (S), and can hinder some molding processes, just like low m.p., which may hinder processing, drying and stability. Differential scanning calorimetry (DSC) or the Kofler method are considered to be the methods of choice for obtaining melting point data, due to their ability to detect additional thermal data. The determination of the melting point of a compound is the means by which it can be classified, and its purity identified. Additionally, the melting point has been associated with log S, so its value for a specific API would be useful for determining the latter’s solubility. For example, comparisons of the melting points of 10 cocrystals of API AMG 517 to their corresponding coformers showed that each cocrystal exhibits a melting point lying between the melting points of AMG 517 and the coformers [52]. A correlation coefficient of 0.7849 was determined, which means that 78% of the variability of the cocrystal melting point can be attributed to the variability of the melting point of each coformer selected, i.e., if a higher melting point for a cocrystal is desired, then a coformer with a higher melting point needs to be selected. In research conducted on 50 cocrystal samples, 26 out of 50 (i.e., 52%) exhibited a melting point between that of the APIs and that of the coformers, 19 out of 50 (i.e., 39%) had a melting point lower than that of the APIs and that of the coformers, three out of 50 (i.e., 6%) had a melting point higher than that of the APIs and that of the coformers, while two out of 50 (i.e., 4%) had the same melting point as the APIs or the coformers. The above considerations indicate that the melting point of an API can be changed through cocrystallization. For example, carbamazepine/nicotinamide (1:1) cocrystal has a m.p. of 151–161 °C, while the raw drug and the coformer exhibit m.p. at 192 and 128 °C, respectively.

**Figure 7:**
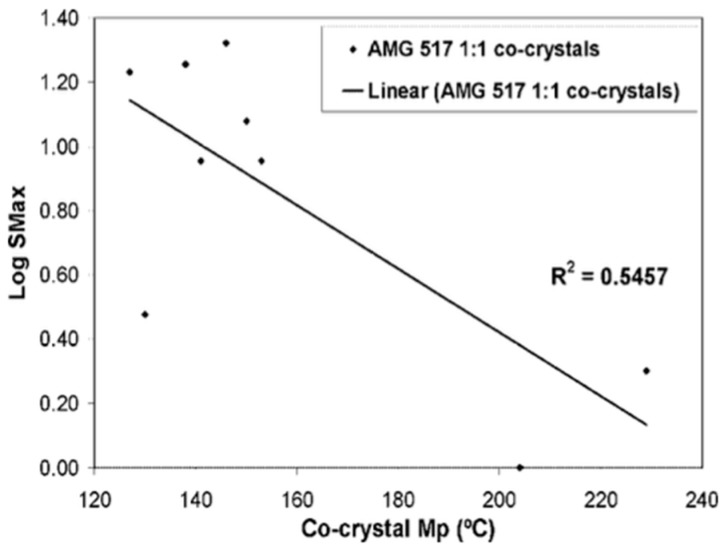
*S*_max_ as a function of cocrystal melting point, showing a 55% correlation. S_max_: the maximum solubility observed in the solubility over time profile. (Reprinted from [30] with permission. Copyright 2013 Elsevier).

In a AMG517 study of the association of solubility (Log *S*_max_) with m.p., 55% of the cocrystals showed association between the *S*_max_ variability and the m.p. variability in the cocrystal, see Figure 7; while in another study on molecules, there was a low association between the cocrystals’ m.p. and the log *S*, which ultimately proved to be a poor indicator of cocrystal solubility [53].

**Figure 8:**
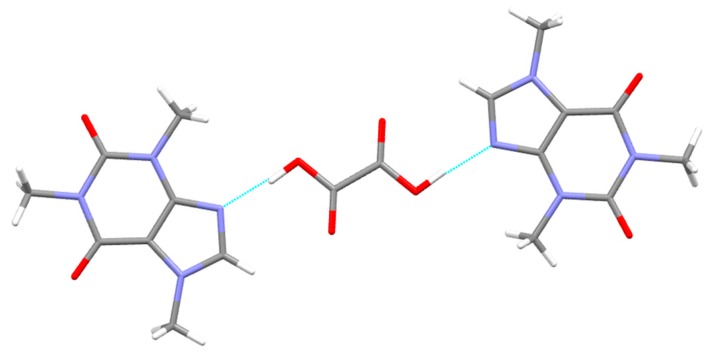
Caffeine/oxalic (2:1) cocrystals with enhanced stability to moisture. Grey: carbon; blue: nitrogen; red: oxygen.

(2) **Stability**: Stability to different types of stress (humidity, heating, light, hydrolysis) is dependent on the structure and characteristics of the API molecule, and is always taken into consideration.

(i) *Relative Humidity (RH)*: In solid forms, changes in RH must be considered when developing a cocrystal. Studies on automated humidity sorption/desorption are usually performed to determine the “problematic” conditions and give directions for more detailed studies, if necessary. Moisture uptake can be controlled through the exposure of the cocrystal to a particular RH using an appropriate humidity chamber and then analyzing the sample after reaching equilibrium. A systematic study in which caffeine was cocrystallized with various carboxylic acids, namely oxalic, malonic, maleic and glutaric acid, showed that the cocrystals produced exhibited reduced hygroscopicity compared to the raw API. The samples were placed in four RH conditions and analyzed after 1, 3 and 7 weeks. The caffeine-oxalic acid (2:1) cocrystals (Figure 8) exhibited complete stability to moisture in all RH conditions [54].

Similarly, for four theophylline cocrystals, the cocrystal with oxalic acid showed greater stability than theophylline [55]. Another example is the indomethacin-saccharin (1:1) cocrystal, which showed minimal water sorption (<0.05%) at 95% RH [56], and the AMG517/sorbic acid (1:1) cocrystal, which also showed minimal water uptake (0.7% water at 90% RH) [57]. Another cocrystal system of glutaric acid and 2-[4-(4-chloro-2-flouorofainoxy)phenyl]pyrimidine-4-carboxamide showed less than 0.08% moisture sorption at 95% RH in repeated sorption/desorption cycles. A long-term study at 40 °C and 75% RH showed no change in the crystal form when assessed by X-ray powder diffraction (XRPD) and DSC analysis [58]. Additionally, the protection from moisture of the carbamazepine/nicotinamide (1:1) cocrystal under conditions of high RH was very significant compared to the anhydrous carbamazepine, and the formation of hydrates of the cocrystal and liquefaction of the nicotinamide was prevented [59]. In conclusion, cocrystals may be more stable under processing and storage conditions.

(ii) *Thermal stress*: Stability (physical and chemical) of the solid API under high temperature conditions is always evaluated. A study examining the cocrystal of a monophosphate salt with phosphoric acid at 60 °C showed no detectable degradation or transitions between forms [60].
(iii) *Photostability:* Carbamazepine undergoes photodegradation, with the mechanism depending on the distances between the rings in the crystal lattice (it requires < 4.1 Å). The carbamazepine-saccharin and carbamazepine-nicotinamide cocrystals have longer ring distances, eliminating the mechanism of photodegradation. Thus, the cocrystal can be protected from unwanted processes, since cocrystallization may affect chemical stability through the rearrangement of the molecules in the crystal lattice [59,61].
(iv) *Solution stability:* This is defined as the ability of the cocrystal components to remain in the solution and to not readily crystallize. This is an important parameter to evaluate during development, both for solutions and suspensions, as well as for solid dosage forms that will dissolve in the gastrointestinal tract. Since cocrystal dissociation may occur, the stability in solution is a key element in their development. A study on carbamazepine cocrystals with 18 coformers evaluated the formation of carbamazepine hydrate when the cocrystals were slurried in water for 24–48 h. Of the studied cocrystals, seven maintained their crystalline structures, and the rest were converted into carbamazepine hydrate. The aqueous solubility of the coformer appeared to be an important parameter for the formation of the hydrate. It was noted that cocrystals containing coformers with relatively high solubility in water resulted in the hydrated form, while cocrystals with coformers of relatively low solubility remained stable in aqueous media [43].

(3) **Solubility**: Solubility can be expressed as saturation or thermodynamic solubility, as dissolution rate (kinetic parameter), and as intrinsic solubility. Saturation or thermodynamic solubility is measured on reaching equilibrium in solution with undissolved substance in a solvent, and is usually determined by a single measurement, generally after 24–48 h [62]. Dissolution rate is based on measurements at selected time points, and intrinsic solubility is a measure of the speed of dissolution in the absence of the influence of the particle size (mg/cm^2^·min).

The first investigation on the behavior of cocrystals in solution as a function of cocrystal component concentration was based on the extensive knowledge of molecular complexes, solid-state complexes, and molecular compounds that existed before the introduction of the term of cocrystal, and is analogous to the effect of common ions on the solubility of sparingly soluble salts [63]. Connors et al. [64] focused on the complexation among the components and their experiments and showed that in cocrystals an increased concentration of coformers leads to a reduction of the drug concentration at equilibrium. Mathematical models describe the solubility of a cocrystal in terms of solubility product (*K_sp_*) and constant of complexation in solution (*K*_11_), which was introduced in the work of Nehm et al. [65].

(1) $$AB_{solid}\begin{array}{c} K_{sp}\\ ⇌ \end{array}A_{soln}+B_{soln}$$

(2) $$A_{soln}+B_{soln}\begin{array}{c} K_{11}\\ ⇌ \end{array}AB_{soln}$$

(3) [A]T=[A]=[AB]

(4) $$\left[A\right]T=\frac{K_{sp}}{\left[B\right]T}+K_{11}K_{sp}$$

The chemical balances that describe the solubility of *AB* cocrystals are given in Equations (3) and (4), where *A* is the drug and *B* is the drug coformer.

Equation (4) is an expression of the solubility of the cocrystals with regard to drug concentration versus concentration of coformer equilibrium, indicating that cocrystal solubility decreases as a function of coformer concentration due to the probability of solution supersaturation. Therefore, the solubility of the cocrystal is directly proportional to the solubility of its ingredients, and increases according to the solubility of the coformer. Rodriguez-Hornedo et al. [66] reported that the coformer’s solubility should be about 10 times higher than that of the API to enhance the solubility of the cocrystals. Studies from the same research group reported dissolution results for 25 carbamazepine cocrystals. Some showed a much higher solubility enhancement; e.g., an enhancement of 152 times for carbamazepine and nicotinamide. The addition of the high-solubility nicotinamide in the crystal lattice of ibuprofen increases its solubility more than 7.5 times [67].

**Figure 9:**
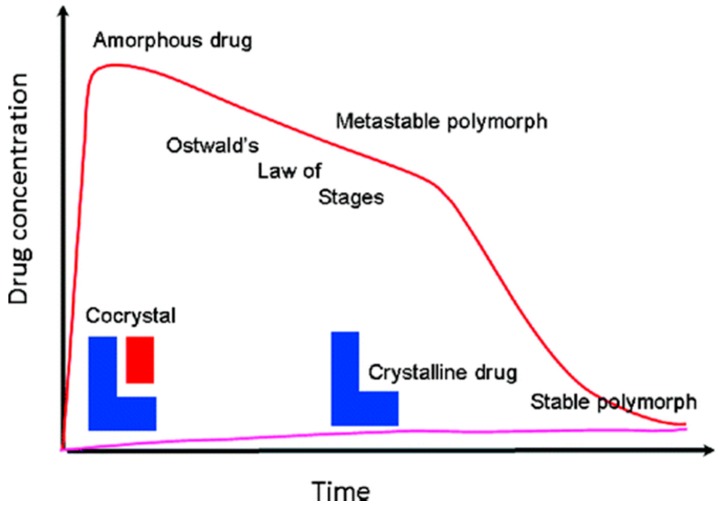
The spring and parachute concept for achieving high apparent solubility with cocrystals. (Reprinted from [69] with permission. Copyright 2011 American Chemical Society).

The dissolution rate of cocrystals increases as a result of increased solubility, as described by the Nernst equation [68]. In most cases, the increased amount of cocrystal that is dissolved occurs in a short time (<30 min) and is maintained for a sufficiently long time (4–6 h). The increased amount of dissolved cocrystals is linked to supersaturation phenomena that are characteristic to amorphous drugs. A hypothetical mechanism that interprets the supersaturated drug delivery systems is known as the “spring and parachute”, and is shown in Figure 9 [69]. This sudden phenomenon may lead to supramolecular aggregates or clusters of randomly oriented molecules without a high-level organization and periodic arrangement similar to the amorphous state of the drug (spring effect). Maintenance of high solubility, or the parachute effect, may last for a substantial amount of time (120–300 min), because the transitions of these amorphous sums to stable crystalline phases and crystal development is expected to be a slow process that is prevented by polymers and excipients that are present in the stomach along with the drug. This high-energy amorphous phase is expected to fall into a metastable polymorphic form of the drug (of higher solubility), and eventually into a stable thermodynamic crystal form following Ostwald’s step rule, but until then, much of the drug will have been absorbed. The solubility of the cocrystals has been reported in a variety of media (water, 0.1 N HCL, phosphate buffer, etc.). In solubility experiments, biorelevant dissolution media such as simulated gastric fluids (SGF) or intestinal fluids (SIF) are also used. Dissolution may be dependent on the particle size.

The influence of the cocrystals’ particle size on dissolution was studied based on observation of carbamazepine/saccharin (1:1) cocrystals dissolved in SGF solution. When the particle size was smaller than 150 μm, faster initial dissolution was observed. Dissolution was virtually complete when the particle size was smaller than 500 μm, while slower dissolution was observed for particles larger than 500 μm. Larger particles (500 μm^−1^ mm) were found, using the XRPD technique, to contain a mixture of cocrystals and carbamazepine hydrate when exposed to SGF fluid overnight, and the slower dissolution observed was attributed to their presence when compared to smaller particles [70]. Therefore, reduction of the cocrystal particle size should be studied and fine-tuned during their development.

With regard to intrinsic solubility, there have been only a limited number of studies on cocrystals. Glutaric acid 2-[4-(4-chloro-2-flouorofainoxy)-phenyl]pyrimidine-4-carboxamide cocrystals were studied by placing them in water for 90 min. Cocrystals’ intrinsic solubility was found to be 18 times higher than the parent compound [58].

(4) **Bioavailability**: Up until now, studies on pharmaceutical cocrystals’ bioavailability, even in animals, have been limited. Bak et al. [57] showed positive results for a 1:1 cocrystal of a AMG517 compound with sorbic acid. Regarding the cocrystal, a ten-fold increase was observed for maximum plasma concentration (*C*_max_) and area under the curve (AUC) compared to an equivalent dose (500 mg/kg) of the crystalline drug in rats. Cocrystals that have shown higher *C*_max_ and AUC in plasma compared to the pure drug include a cocrystal of Merck L-883555 and L-tartaric acid (1:2) in monkeys (15-times higher bioavailability) and an indomethacin-saccharin (1:1) cocrystal in dogs [71,72].

**Figure 10:**
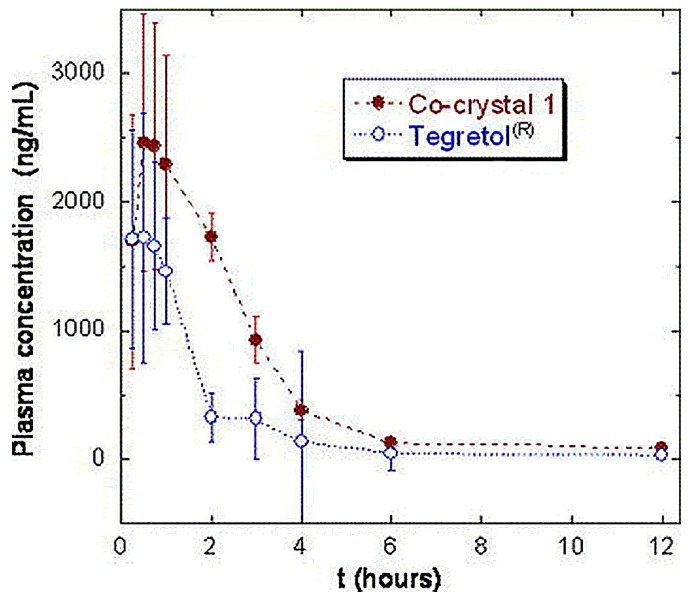
Average plasma time curves of carbamazepine concentrations (±SEM) from a cross-over experiment in fasted beagle dogs (n = 4) given oral doses of 200 mg of the active drug as Tegretol^®^ tablets and as cocrystals. (Reprinted from [70] with permission. Copyright 2007 Elsevier).

However, not all attempts to select cocrystals with improved pharmacokinetics compared to their respective parent drugs have been successful. For instance, carbamazepine-saccharin (1:1) cocrystals were administered to dogs and compared to the commercially available immediate-release tablets of carbamazepine (Tegretol^®^). Four dogs were used, and samples were taken 12 h after administration, see Figure 10. Cocrystals with particle size <53 nm were mixed with lactose in a dry mixing step and were placed in capsules containing a 200-mg dose of carbamazepine. The cocrystals appeared to have a better dissolution profile, but no statistically significant differences were reported between the pharmacokinetic parameters of the cocrystal and the marketed product after 12 h [70].

This study showed that cocrystals can be used as a model to address physicochemical problems of a pharmaceutical compound (i.e., stability, solubility, dissolution), without solving, nevertheless, problems of metabolism or pharmacokinetics. A pharmacokinetic study of lamotrigine/nicotinamide (1:1) showed lower *C*_max_ and AUC in plasma compared to the parent drug, even though the powder dissolution rates in water in an acidic environment were comparable. This is due to the fact that the influence of pH and counterions, additives or excipients on the cocrystal solubility and thermodynamic stability under different conditions, such as those between in vitro and in vivo dissolution studies, was not considered [73].

(5) **Mechanical properties**: Mechanical properties, such as tensile strength, forces to rupture, elastic properties, compressibility and tableting capacity may be altered through cocrystallization due to changes in the crystalline structure. For instance, paracetamol cocrystals with different coformers were found to exhibit enhanced mechanical properties [74]. Specifically, paracetamol cocrystals with 5-nitroisophthalic acid were able to be successfully manufactured into tablets of the desired tensile strength without compromising the dissolution profile due to the presence of slip planes in the cocrystals [75]. Other examples of cocrystals with improved mechanical properties include vanillin isomers and the cocrystal of carbamazepine with nicotinamide [76,77].

(6) **Cocrystal polymorphism**: During the development of cocrystals, efforts are made to identify and characterize the polymorphic forms of the compounds because they may have different physicochemical properties. It has been traditionally suggested that polymorphism is a phenomenon seen less frequently in cocrystals than in monocomponent crystals, and that cocrystallization can be used to prevent polymorphism [78]. However, many recent examples of cocrystal polymorphism have been discovered and entered into the CSD [79]. For example, Prohens et al. [80] recently discovered nine new cocrystals of agomelatine, which is an atypical antidepressant. Two of the coformers produced polymorphic cocrystals during screening, indicating that, similarly to single-component crystals, polymorphism in cocrystals should be studied, as it could expand the cocrystal landscape.

## 7. Methods of Cocrystal Preparation

The process of cocrystal formation is not fully understood. It is not clear whether formation of intermediate states (e.g., amorphous phase) precedes cocrystallization. A recent study based on solid-state NMR suggests that cocrystallization is not mediated by the transient formation of an amorphous phase [81]. Until the cocrystal formation mechanism is fully elucidated, cocrystallization methods will remain largely empirical in nature. Effective cocrystal preparation methods in use today can be classified as: (1) solid (grinding, solvent-assisted grinding, sonication) and (2) solvent-based (slurring, solvent evaporation, crystallization from solution or active cocrystallization and antisolvent addition). For the solvent-based methods, the selection of the solvent is crucial, since its potential change will alter the intermolecular interactions and potentially lead to better cocrystallization results. Other methods may also have constraints, such as:

- Thermal methods that require melting need high temperatures, which can affect the integrity of heat-sensitive compounds.
- Mechanical methods, such as grinding, require energy consumption and can produce amorphous materials, limiting their effectiveness if a suitable solvent is not used.
- Methods based on precipitation from solution require continuous and precise control of the supersaturation level of the components’ concentration and necessitate the creation of phase diagrams, while the use of a solvent is not environmentally friendly.

Kuroda et al. [82] suggest that shearing and molecular diffusion during grinding can generate a different additive structure, while vapor diffusion has also been proposed as a mass transport mechanism during the grinding of the solid state [83]. In solvent-based methods, it is necessary to control several variables to select the conditions for the generation of the nuclei and the cocrystal development, in addition to the selection of the solvent. For instance, cocrystal nucleation depends on parameters such as concentration, temperature, cooling rate and rate of evaporation.

**Figure 11:**
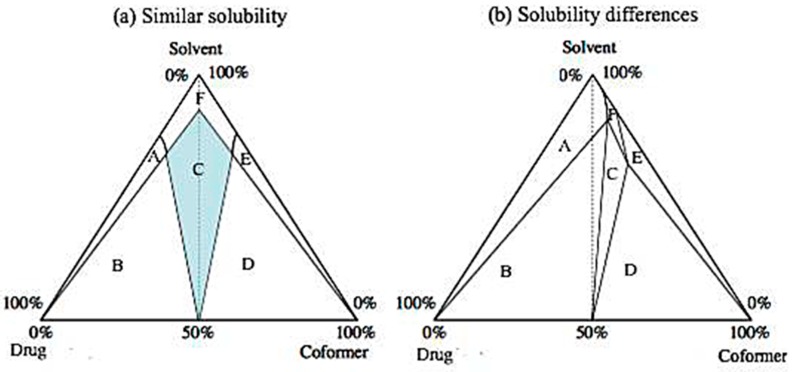
Schematic representations of isothermal ternary phase diagrams with (**a**) similar solubility between drug and coformer in solvent and (**b**) different solubility of drug and coformer in solvent. Region A, drug and solvent; B, drug and cocrystal; C, cocrystal; D, coformer and cocrystal; E, coformer and solvent; F, solution. (Adapted from [87]).

The most common method of industrial production (on a large scale) of cocrystals is the cocrystallization of an API by a supersaturated solution in the presence of a coformer. In most cases, about 40% supersaturation is achieved through the slow cooling of an undersaturated mixture until the dissolution limit is reached. Additionally, solution mediated phase transitions (SMPT) can be induced by manipulating the amount of the coformer (reaction cocrystallization). Specifically, addition of nicotinamide in dry form has been shown to allow the formation of carbamazepine cocrystal [84], and the addition of a certain amount of one of the components of the cocrystal system has been used as a retreatment process in cases where the cocrystal phase was polluted by another crystalline phase (coformer or API) [85]. The key to the composition of high-purity cocrystals is the understanding of the binary or ternary phase diagram for the equilibrium, which includes the solvent (if there is one) and the two components [38]. The binary phase diagram of the two cocrystal components (the drug and the coformer, in the case of pharmaceutical cocrystals) shows the eutectic points between each phase, and thus the existence and the number of the phases of the cocrystal. Ternary phase diagrams are affected by the relative solubility of the two components. If the solubility of the two components in a given solvent is similar, they are presented as in Figure 11a, while Figure 11b refers to components with very different solubilities. In these diagrams, the slow evaporation of a 1:1 solution of the two components may lead either to a cocrystal or to a mixture of cocrystals and the individual constituents. This depends on whether the crystallization path passes through the mixed-phase region or the single-phase region [86].

**Figure 12:**
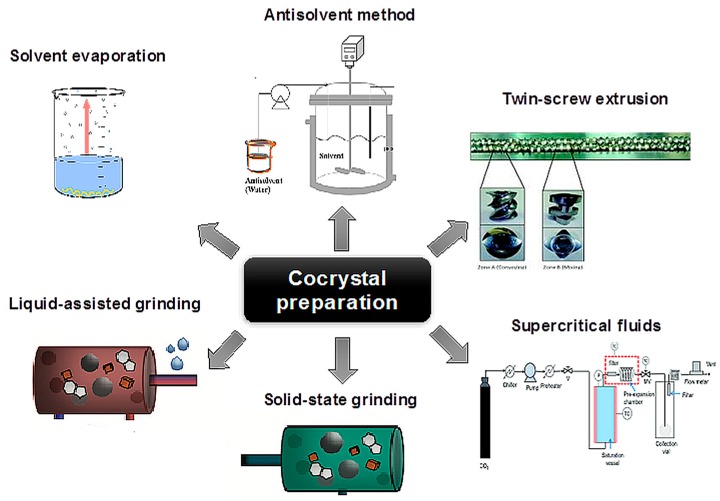
Schematic presentation of methods applied in cocrystal formation.

Chadwick et al. [88] studied cocrystal formation at room temperature, and stressed the importance of the intermediate metastable eutectic liquid phase, which facilitates the transport of intramolecular mass leading to cocrystallization. Based on these studies, they concluded that systems with eutectic temperatures close to ambient temperature are prone to the formation of cocrystals through dry grinding, while in those with eutectic temperatures above the ambient temperature, the addition of solvent helps cocrystal formation. Shan et al. [89] further explained that grinding in the presence of a few drops of solvent results in a system with increased degrees of freedom. The increase in molecular collision facilitates the initial formation of cocrystal nuclei. A quick description of the methods applied in pharmaceutical cocrystal formation follows, apart from the slow cooling of an undersaturated solution and the solution mediated phase transitions (reaction cocrystallization) described above (Figure 12).

(1) *Solvent evaporation technique:* This is the most commonly used technique for generating cocrystals. The materials (API and coformer) are dissolved in a common solvent with a suitable stoichiometric ratio and completely evaporate [90]. During evaporation, the solution of the molecules undergoes changes, with the creation of hydrogen bonds between different functional groups, thus producing a thermodynamically favored product. The selection of the solvent plays an important role in solubility. If the solubility of the two components is not similar, then the component with the lower solubility will precipitate. Preparation of cocrystals by solvent evaporation is a small-scale technique that does not require complex equipment, and results in cocrystals of high quality and purity. However, the use of large amounts of solvent and its limited scalability are two disadvantages to this technique [91].

(2) *Solid-state grinding technique or neat grinding:* This is a cocrystallization method without a solvent. The solid materials that will result in the cocrystal are admixed in appropriate stoichiometric amounts, pressed and crushed together with a mortar and pestle, or a ball mill or vibrator mill. The common grinding duration ranges from 30 to 60 min. With this method, numerous cocrystals can be prepared, and any failure is generally due to the use of inappropriate settings.

Reducing the particle size increases the specific surface area of interaction between the materials for the development of intermolecular bonds. This offers the advantage of increased selectivity compared to cocrystallization through dissolution. It is simple, and allows quick preparation of the desired cocrystal. Experiments on mixing cocrystals with other components that can also form cocrystals with the API have been carried out. In the latter case, the coformer is replaced, and this can be used either to assess the stability of a cocrystal in the presence of other excipients or to disclose alternative modifications of the cocrystals. Modifications that don’t necessarily take place in the process of dissolution, e.g., caffeine-trifluoroacetic acid cocrystal, were initially only obtained by grinding [92]. That is to say that it has also been used as a method of clarifying hydrogen bond preference. Mechanochemistry (i.e., solid-state grinding) was used for the patent of the pterostilbene-caffeine cocrystal [93].

(3) *Liquid-assisted grinding, or solvent-drop grinding:* This is a modification of neat grinding by adding a small amount of solvent during the grinding process, and has been used to enhance supramolecular selectivity, both polymorphic and stoichiometric, in crystalline systems [20]. It includes mixing the two components and adding a very small amount of solvent (~a few tenths of an equivalent of solvent per mole of the component). The effect of the solvent can be described as catalytic, as its small amount is not part of the final product. Its advantages lie in its increased performance, in the ability to control the production of polymorphs, and in the improved crystallinity of the product, while a large number of coformers are suitable for the cocrystallization. This method enhances the cocrystallization rate, as some cocrystals showed poor performance in cocrystal formation following neat grinding for a considerable amount of time [89]. This method can be used to prepare high-purity cocrystals with a significant reduction in the preparation time. It also allows the synthesis of selective polymorphic forms of cocrystals. For instance, in caffeine-glutaric acid (1:1) cocrystals, neat grinding resulted mainly (not always) in form I, while liquid-assisted grinding to pure form I with less polar solvent (e.g., cyclohexane or hexane) and to pure form II with more polar solvent (e.g., water or acetonitrile) [94]. This allows interconversion between crystalline forms of polymorphic organic components, depending on the polarity of the solvent. Limitations of liquid-assisting grinding include the fact that it is a small-scale technique, requires high energy consumption, and has a low performance in terms of product purity. Liquid-assisted grinding was used for the patent of pterostilbene-carbamazepine cocrystals [93,95].

(4) *Slurring technique:* This is a simple process, whereby crystallization solvent is added [96]. The solid API dissolves in the solvent, forming a solution into which the coformer is added, after which the resulting suspension is stirred, filtered and dried. Slurring was used for the patent of celecoxib-venlafaxine cocrystals [97]. This multi-drug cocrystal combines the therapeutic properties of celecoxib (which has anti-inflammatory properties for patients with chronic musculoskeletal inflammatory diseases) and venlafaxine (with an antidepressant effect).

(5) *Antisolvent cocrystallization:* a solvent in which the compound is less soluble is often added to the solution, favoring the precipitation of the solids. The resulting suspension is filtered, and the collected solid can be characterized by XRPD. Disadvantages of this method are its lower performance compared to grinding that uses a solvent, as well as the large volume of solvent used. Antisolvent crystallization has been reported for the production of carbamazepine-saccharin and indomethacin-saccharin cocrystals [98,99]. In both studies, the construction of phase solubility diagrams was an integral part of the methodology for identifying the optimal conditions (e.g., ratio of solvent to antisolvent) for the formation of cocrystals.

(6) *Use of supercritical fluids:* Supercritical fluid (SCF) is a very good solvent, and has the unique ability to diffuse through solids like a gas and dissolve materials like a liquid (gas flow properties and dissolving liquid properties); thus, it can replace organic solvents. CO_2_ is the most frequently used supercritical fluid for cocrystallization as solvent, as anti-solvent and as atomized anti-solvent [100]. A detailed review of the preparation of pharmaceutical cocrystals through sustainable process using supercritical carbon dioxide has been provided by Pando et al. [101].

(i) *Cocrystallization with supercritical solvent (CSS):* The active substance and the coformers are dissolved in the supercritical CO_2_ (sc-CO_2_) inside a stainless-steel vessel, and depressurization then leads to the loss of dissolving dominance of sc-CO_2_, to supersaturation and eventually to the formation of cocrystals. The application of CSS requires sufficient (ideally equal) solubility of the pure components in sc-CO_2_. Its main disadvantage is its low performance in pure products.
(ii) *Supercritical antisolvent (SAS):* If a substance is not soluble in sc-CO_2_, the sc-CO_2_ can be used as an anti-solvent for a solution of cocrystal components (coformer and API) in an organic solvent. Therefore, the active substance and the coformers dissolve into an organic solvent (primary solvent). This is followed by its dropwise mixing with the sc-CO_2_ by passing the organic solution through a nozzle. The sc-CO_2_ dissolves quickly in the droplets of the organic solution, reduces the dissolving power of the solvent and simultaneously extracts it, causing saturation and supersaturation, during which cocrystallization nuclei are formed and the precipitation of cocrystals by the anti-solvent effect of sc-CO_2_ takes place. This technique requires complete miscibility of the organic solvent with the sc-CO_2_ and lower solubility for the solute in the mixture. Then, the organic solvent is removed, and a product without solvent is obtained. Itraconazole (antifungal drug of poor bioavailability) and succinic acid cocrystals preparation, with sc-CO_2_ as an antisolvent, is an example of such [102], while the same method was used for the patent of the carbamazepine-aspirin cocrystal [103].
(iii) *Atomized anti-solvent (AAS):* In the AAS technique, the sc-CO_2_ enhances the atomization of the organic solution, producing particles by two different mechanisms: antisolvent crystallization and spray-drying crystallization. The solution containing the API and coformer is pumped through a coaxial nozzle, where it mixes with the sc-CO_2_ or N_2_ in the mixing chamber prior to its depressurization into the precipitator vessel (for SAS technique, the precipitator is filled with CO_2_ at high pressure, whereas in the AAS technique, it is at ambient pressure). Pure indomethacin–saccharin cocrystals have been produced by the AAS technique [100].

(7) *Sonocrystallization:* Ultrasound, apart from its wide application in various fields of medicine (e.g., as a diagnostic method) and cosmetology, offers promising prospects in the generation of nuclei during the process of crystallization of drugs. For example, it has been used for the formation of pharmaceutical microparticles sized 2–6 μm, primarily intended for inhalation [104]. The generation of such particles through mechanical methods causes problems of physicochemical stability, performance, and product modification, attempts to improve all of which are being made through the use of the escalating power of ultrasound.

When the ultrasound waves pass through the inner part of the liquid solution with alternating cycles of high pressure (compression)–low pressure (thinning), they create air bubbles or voids in the liquid. The volume of the air bubbles increases due to the absorption of the supplied energy of the ultrasound. However, when supersaturation occurs and they cannot absorb any more energy, during a high-pressure cycle, they collapse in the liquid violently and the temperature increases due to the release of the energy of the bubbles. This collapse is referred to as cavitation. The content of the air bubbles is quickly compressed. This results in the promotion of crystallization and precipitation, since homogeneous mixing of API and coformer has already been achieved. At the moment of total collapse, the vapor’s temperature can reach ~50,000 K and its pressure ~200 atm. The whole energy supply to the liquid is continuously monitored through the ultrasonic monitor. Thus, API and coformer are added in the appropriate solvent in similar stoichiometric proportions, and their solution is generated, which is subsequently sonicated. The ultrasonic waves are of high intensity, ~20 KHz, and promote the formation of crystallization nuclei.

Cocrystal formation under sonication depends on several parameters, such as the solvent, the time of sonication, the saturation levels of the APIs and the coformer. The sonocrystallization technique was used for the patent of the hydrochloride fluoxetine cocrystal with benzoic acid in acetonitrile [105].

(8) *Intermediate phase or form modifications:* Generating cocrystals requires a process of modification of the phases or forms of the API and the coformers (e.g., melt, solution, polymorphic transition, etc.) to achieve successful cocrystallization. The required modification may pass through consecutive intermediate stages, not all of which are always easily identifiable, because the transitions are very fast, no matter which process is used (e.g., grinding, solvent grinding, melt cooling, moisture sorption). The intermediate forms, such as hydrates, amorphous form or metastable polymorphic form, act as unstable intermediates in the cocrystallization nucleus [106]. However, in this method a thorough examination of the thermodynamic properties of the compounds is necessary.

Intermediate phases or forms that have been identified have, until recently, been elucidated by interruption of the process, e.g., stopping the grinding and testing the product for new solid forms (amorphous, coamorphous, polymorphs, cocrystals, salts). These data have not always been reliable as a result of the possible moisture sorption and recrystallization that can occur during testing, since the examination has not taken place in real-time (in situ).

**Figure 13:**
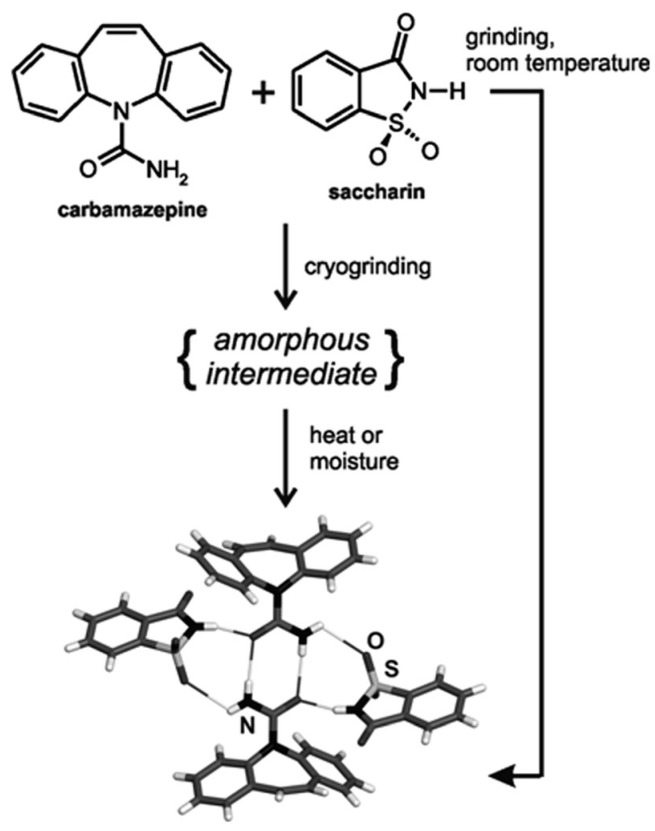
Mechanochemical cocrystallization by neat grinding of carbamazepine and saccharin, involving an amorphous intermediate. (Reprinted from [109] with permission. Copyright 2012 Royal Society of Chemistry).

**Figure 14:**
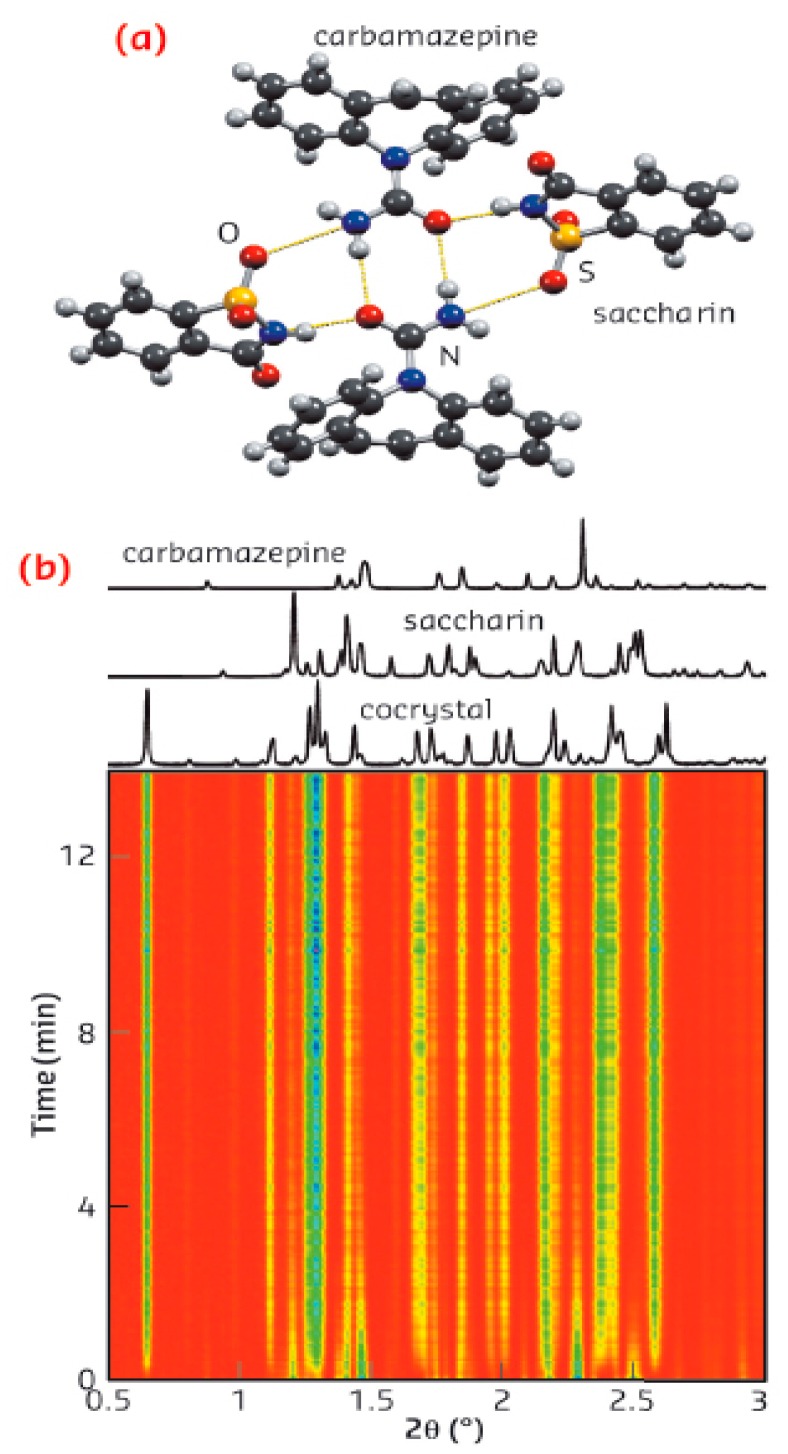

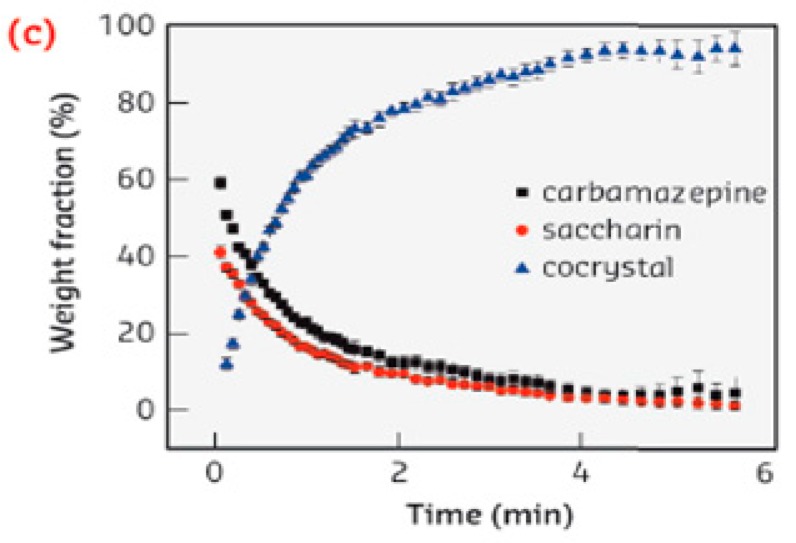
(**a**) Fragment of the crystal structure of the pharmaceutical cocrystal of carbamazepine and saccharin. (**b**) Time-resolved X-ray diffractogram for the cocrystallization of saccharin and carbamazepine using liquid-assisted grinding. (**c**) Reaction course obtained via Rietveld analysis. (Adapted from [107] with permission. Copyright 2013 Willey).

A contemporary technique for direct and real-time (in situ) monitoring of transformations during the process has been carried out using X-ray diffraction. The first application through intermediate amorphous phase was in a carbamazepine-saccharin mixture (Figure 13 and Figure 14). The difference between neat and liquid-assisted grinding was found to be significant, as the latter creates cocrystals very quickly (in 2 min) compared to neat grinding, where amorphous intermediate phases could be observed [107]. This method provided, for the first time, a rapid in situ process of cocrystallization, as well as the study of several intermediate phases that had not previously been observed. Intermediate form modifications were used for the patent of generating choline cocrystals, which protect against diabetic complications and provide cardioprotection for non-diabetic patients, too [108].

**Figure 15:**
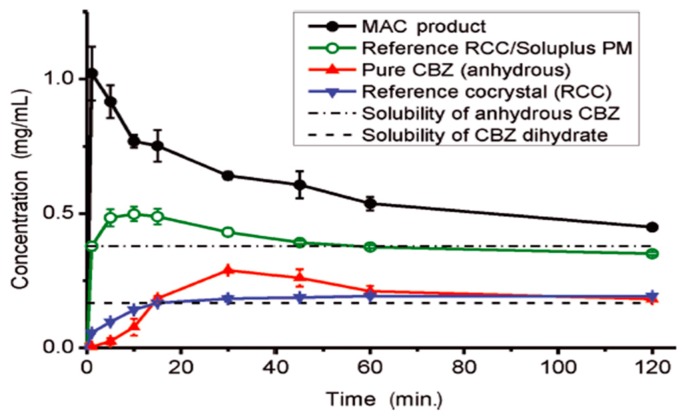
Comparison of in vitro dissolution profiles among CBZ-SAC/Soluplus^®^ formulations. CBZ: carbamazepine, SAC: saccharin and PM: physical mixture (Reprinted from [116] with permission. Copyright 2014 Elsevier).

(9) *Spray drying*: This method has been used recently for generating cocrystals [110]. Solutions of disparately saturated systems lead to the formation of pure cocrystals, as opposed to the mixtures obtained after solvent evaporation. Thus, cocrystal formation can be monitored kinetically and/or mediated by the glassy state of the material.

(10) *Resonant Acoustic Mixing^®^ (RAM):* This is a non-contact mixing technology that relies upon the application of a low-frequency acoustic field to facilitate mixing. This new technology has shown several advantages for numerous complexes and multiphase systems, while it reduces both mixing time and cost. Carbamazepine-nicotinamide cocrystals have been formed with this method by adding a solvent (20 μL/100 mg of the cocrystal component) such as CH_3_Cl, H_2_O, DMF, DMSO, MeOH, and LabRAM was operated for two hours at 90% intensity with automatic tuning [111]. This method can be used for cocrystal production at a large scale, and provides high-purity cocrystalline products.

(11) *Twin-screw extrusion (TSE)*: Extrusion is the process of converting an unprocessed material or mixture of materials that have been ground or granulated into a product of uniform shape and density by pressing them through a die under controlled conditions [112]. During TSE, the mixture of the active substance, the thermoplastic polymer carrier, excipients, and other auxiliary agents (e.g., plasticizers, antioxidants), is heated in the extruder [113]. By the end of the process, a solid form (e.g., granules) is obtained from the output of the extruder. TSE was used for the patent of the L-malic acid and L-tartaric acid cocrystallization [114]. Medina et al. [115] showed that the TSE technique can be used as an effective method for generating pharmaceutical cocrystals. Furthermore, the matrix-assisted cocrystallization (MAC) approach uses the TSE technique to produce cocrystals embedded in a formulation matrix. Equimolar amounts of API and coformer are mixed in solid form with a matrix material prior to the feeding in the extruder. The extruder is set at a temperature where only the matrix is liquid via either the formation of soft material or melt. Cocrystallization occurs during extrusion due to the mixing and grinding of the components in the pliable matrix. The cocrystal particles formed in this way are incorporated in the matrix, where they remain in a fluidized state until the exit from the extruder. In the MAC product, the material of the matrix plays a double role: (1) during TSE it plays a role similar to that of a catalyst, for example, as a solvent. The use of a molten or pliable matrix promotes good mixing and reduces the excessive shear stress generated when solid materials are placed in the extruder. This reduction in the shear stress mitigates potential damage to the crystalline structure; and (2) the matrix acts a functional component of the formulated product in the finished product. Thus, the selection of the matrix material is very important for offering additional functionality to the formulated cocrystal, such as improved flowability, compressibility and release kinetics of the drug, Figure 15 [116]. The MAC method provides simultaneous production and formulation of the pharmaceutical cocrystal, giving high-quality cocrystals.

## 8. Characterization of Cocrystals

Several methods have been applied for the characterization of pharmaceutical cocrystals and the elucidation of intermolecular interactions. Recently, Pindelska et al. [117] provided a detailed review on the current advances in the characterization of pharmaceutical cocrystals, salts and polymorphs, from bulk down to the molecular level. The final section of this review covers in brief techniques for the characterization of the cocrystals, especially those that are routinely used in drug delivery and development laboratories.

(1) *Single-crystal and powder X-ray diffraction (XRD):* XRD methods comprise the predominant tool used for the characterization of cocrystals. Single-crystal XRD is routinely used for the structure solution of cocrystals, while powder XRD (PXRD) is mainly used for identification purposes, since cocrystals exhibit characteristic sharp peaks that are different from the peaks of the cocrystal components. Moreover, quantification of cocrystals in the crystallization mixture using PXRD has been reported by Pardela et al. [118], who developed and employed this method to study the formation of indomethacin-saccharin cocrystals by mechanochemistry. Currently, software programs such as DIFFRAC.TOPAS (Bruker AXS, Karlsruhe, Germany) allow structural determination and refinement based on Rietvield analysis. They are also routinely used to assess the yield of cocrystallization by being able to quantify the percentage of cocrystals and their components in a mixture. A variety of thermal and spectroscopic techniques are used simultaneously to characterize and quantify potential new cocrystals.

(2) *Thermal analysis*: Thermal analysis refers to a group of techniques that record the physical or chemical changes to the sample thermal properties via programmed temperature change (e.g., heating, cooling, alternating, or maintaining at a constant temperature) versus time and in a controlled atmosphere. Mass, heat or heat flow, enthalpy, etc. can be the measured properties. For cocrystal characterization, thermogravimetric analysis (TGA), differential thermal analysis (DTA) and differential scanning calorimetry (DSC) are the most applicable, together with hot-stage microscopy (HSM). Below, a short introduction to the use of DSC and HSM for the characterization of cocrystals is given:

(i) *Differential scanning calorimetry (DSC)*: For cocrystals, DSC is useful for the construction of binary phase diagrams in the screening of cocrystal formation or the existence of a eutectic mixture or eutectic impurities, which reduce the melting point [119]. It measures the heat of fusion, heat of transition in solid-solid transitions, and heat capacity. It can also be used for the determination of the degree of crystallinity (measurement of enthalpy of fusion of the sample and comparison to the value of the fully crystalline material).

Binary phase diagrams show the formation and the stability of the generated cocrystal. The cocrystal’s melting point differs from the melting points of the individual components—sharp endothermic peaks appear at lower temperatures in comparison to the sharp endothermic peaks at higher temperatures of the individual components. In cases where substances exist as impurities, a second peak can be created as a continuation of the main melting peak. The existence of cocrystals is proved via DSC at a melting temperature that is usually between the melting temperatures of the pure compounds. It is also used for in situ cocrystal formation. When a mixture composed of two ingredients able to form a cocrystal is heated, an exothermic peak associated with cocrystal formation is detected immediately following an endothermic peak. In combinations that do not give cocrystals, only one endothermic peak associated with the eutectic point will be detected [42].

**Figure 16:**
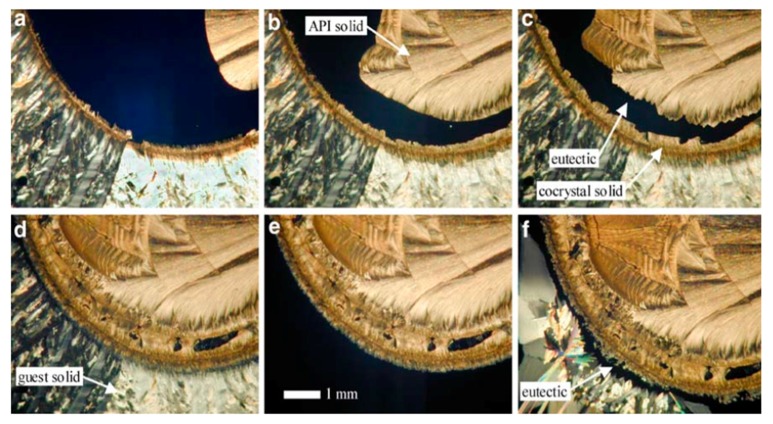
Cocrystal screening using the Kofler technique under a hot-stage microscope. Images (**a**–**c**) depict the growth of the cocrystal (brown curved zone in center) at the boundary of the glutaric acid. The dark area corresponds to the melting of the eutectic formed by the cocrystal and the API. In (**d**) a narrow dark area corresponds to the melting of the eutectic formed between the cocrystal and the glutaric acid. Image (**e**) depicts complete melting of the glutaric acid, making the boundary with the cocrystal clearly visible. Image (**f**), shows growth of the glutaric acid with formation of eutectic with the cocrystal, following cooling of the melt. (Reprinted from [58] with permission. Copyright 2006 Springer).

DSC has been used in the study of the cocrystal formation mechanism, such as the study of the kinetics of the in situ formation of the carbamazepine-nicotinamide cocrystal from equimolar components in amorphous state after melting [120]. The amorphous state was formed by heating to above 158 °C, melting and cooling to −30 °C at a constant rate in the DSC or in a heated optical microscopy slide. The molecular mobility of the amorphous state can lead to molecular cocrystal formations. Rapid-heating DSC was used to isolate and characterize the metastable carbamazepine-nicotinamide cocrystal form II, formed in situ via heating at a rate of 500 °C min^−1^ [121].

(ii) *Hot-stage microscopy (ΗSΜ):* HSM is a combination of microscopy and thermal analysis to study the physical characteristics of materials in solid form as a function of temperature and time. When the drug crystals are heated, they undergo changes that can be quickly and easily observed through the microscope. In this way, thermal changes such as melting point, melting range, crystal growth, crystalline transformations, etc. can be visualized. HSM is a simple and relatively inexpensive technique. The systems of HSM allow the controlled heating of the sample, which is placed on a glass slide and viewed under the microscope. Heating is achieved by heat transfer from a metal element, which is heated thermoelectrically. The HSM instrument can be combined with other devices, such as Fourier-transform infrared spectroscope (FTIR), DSC, or a heating-cooling system for regulating the flow of hot or cold air. An important application of thermal microscopy, besides those reported by Stieger et al. [122], is the in situ formation of cocrystals, which is also known as the Kofler contact preparation method (Figure 16) [123].

(3) *Spectroscopy:* Spectroscopic methods that can be used for the characterization of cocrystals belong to two kinds; namely, vibrational spectroscopy and nuclear magnetic resonance (NMR). Vibrational spectroscopy can be further distinguished as belonging to either absorption (infrared, IR) or scattering (Raman) methods. The infrared range can be classified as mid IR (4000–400 cm^−1^), near IR (NIR: 14,000–4000 cm^−1^) and far IR (400–10 cm^−1^). Below, the use of IR and Raman spectroscopy for the characterization of cocrystals is presented. NMR is a powerful characterization tool that can provide detailed information on the structure of organic pharmaceutical cocrystals and complexes. Insightful studies on this complex characterization tool have been provided by Vogt et al. [124] and Pindelska et al. [117].

Regarding Fourier-transform IR (FTIR), the simultaneous study of the spectra of the cocrystals’ individual components and of their final mixture with polymer matrices, etc., is an important tool in detecting cocrystal formation and in the elucidation of their structures. The cocrystal provides a different spectrum from that of the components’ mixture due to the presence of hydrogen bonds, especially when carboxylic acid is used as a coformer and when a neutral hydrogen bond O–H ⋯ N is formed between an acid and a base. Clear IR spectra differences are observed between a neutral carboxylic acid functional group and a carboxylic anion. Neutral carboxylate (–COOH) shows a strong tension band of C=O at about 1700 cm^−1^ and a weaker tension band of C–O at about 1200 cm^−1^, while the carboxylic anion (–COO–), due to coordination, shows a tension band of C–O in the fingerprint region (1000–1400) cm^−1^. If a neutral hydrogen bond O-H ⋯ N is formed between the components, then two broad zones will be observed at about 2450 cm^−1^ and 1950 cm^−1^ [125]. Strong hydrogen bonds are formed in (ΝΗ–Ο), (ΟΗ–Ο), (–ΝΗ–Ν), and (ΟΗ–Ν), while weak ones are formed in (–CH–O) and (CH–O=C).

IR is sensitive to changes in the crystalline form, to polymorphisms and to cocrystal detection. To quantify cocrystals by IR becomes difficult, due to the strong absorption of excipients or other sample components, since most excipients form bonds with a high dipole moment, and numerous absorptions appear that cannot easily be separated and assigned to each component [126]. Instead, NIR has been used as an analytical technique during the process for the monitoring of ibuprofen-nicotinamide cocrystal formation via a rotating dual-screw extruder as a real-time process technique [127]. The technique was considered to be sensitive due to the appearance of new peaks in the region 4800–5200 cm^−1^, and it was eventually proposed for the control of the purity of cocrystals on an industrial scale. However, Raman shows better accuracy and sensitivity, and has been used for formation evaluation and in situ monitoring of cocrystallization either by itself or in combination with NIR [128,129].

Raman spectroscopy has been used in monitoring the crystallization process. It is a valuable analytical method for differentiating between polymorphs, salts, cocrystals, solid solutions and hydrated salts, because it requires minimal sample preparation, and uses only a small amount of it [130]. Furthermore, it is a non-destructive technique for the characterization of compounds, since the intensity of the Raman scattered radiation is low.

Specifically, for cocrystallization, the Raman spectra are useful in the evaluation of cocrystal formation, as the oscillations of the cocrystals are different from those of the starting materials [131]. They also assess whether the researched complex is a cocrystal or a compound in ionized state. The characteristic zones that correspond to the bending and stretching vibrations of amino and carboxyl groups are shifted to lower frequencies when they form cocrystals, and any increase in bond length is due to hydrogen bonding.

Fourier-transform Raman (FT-Raman) is particularly important for the identification and quantitative analysis of polymorphic forms and cocrystals, as it requires very little preparation of the sample and thereby reduces the risk of transition in a metastable polymorphic form, while the spatial resolution is easier and sample format have little effect. Nevertheless, Raman also presents certain restrictions. For example, signal intensity may be restricted by the particle size [132], while fluorescent samples, which can absorb heat, result in low quality spectra due to the background and can even burn the sample or cause polymorphic transition (in loco). Of course, there are certain procedures that can reduce these problems. For example, by using the average spectrum resulting from spectra obtained from different locations of the sample. The problem of the large cocrystals is solved by using a mortar when the API is not converted into another polymorphic form upon the application of pressure [133]. Ibuprofen and nicotinamide cocrystals were formed with supercritical fluid, molded and characterized quantitatively by different analytical solid-state techniques. DSC and Mid-IR techniques were insufficient for quantifying the components of the mixture. The average prediction error was about 15%. Instead, PXRD and Raman techniques showed the best results. Raman, in conjunction with the partial least squares (PLS) multivariate model, was able to predict the concentrations of cocrystals and coformer quickly and accurately. Showing a relative error of less than 5% for the prediction of the concentration, these techniques led to a calibration model to estimate the purity of the cocrystal after its synthesis. This was the first quantitative analysis of the ibuprofen cocrystal together with the coformer [134].

## 9. Conclusions

Pharmaceutical cocrystals possess a high potential for API physical and biopharmaceutical property enhancement, and therefore constitute a field of study that is currently experiencing rapid development. Several methods are available for their formation and physicochemical characterization, and attempts are being made at developing theoretical as well as empirical methods for the prediction of cocrystal formation; attempts that have been hindered, so far, by the lack of sufficient understanding of the mechanism of cocrystal formation. As our understanding of the intermolecular interactions that define their formation and stability increases, theoretical methods for the prediction of cocrystal formation could become an important tool for aiding cocrystal-based dosage form design. Additionally, the development of industrially applicable methods for their production, together with the development of a clear and favorable regulatory framework, is expected to increase their importance as alternative approaches to salt formation or API polymorph selection in the near future.
